# Biomechanical evaluation of annulus fibrosus repair with scaffold and soft anchors in an ex vivo porcine model

**DOI:** 10.1051/sicotj/2018020

**Published:** 2018-09-07

**Authors:** Kresten Rickers, Michael Bendtsen, Dang Quang Svend Le, Albert Jvan der Veen, Cody Eric Bünger

**Affiliations:** 1 OrthopeadicResearch Laboratory, Aarhus University Hospital, Aarhus Denmark; 2 VU University Medical Center, Department of Physics and Medical Technology, Research Institute MOVE, Amsterdam The Netherlands

**Keywords:** Annulus fibrosus, Scaffold, Intervertebral disc herniation, Soft anchors, Suture, Biomechanics

## Abstract

*Introduction*: Altered biomechanical properties, due to intervertebral disc (IVD) degeneration and missing nucleus fibrosus, could be thought as one of the reasons for the back pain many herniation patients experience after surgery. It has been suggested to repair annulus fibrosus (AF) to restore stability and allow nucleus pulposus (NP) replacement and furthermore prevent reherniation. The aim of this study was to evaluate a new method for closing a defect in AF for use in herniation surgery.

*Methods*: Our repair method combines a polycaprolactone (PCL) scaffold plugging herniation and soft anchors to secure the plug. Ex vivo biomechanical testing was carried out in nine porcine lumbar motion segments. Flexion–extension, lateral bending and rotation were repeated three times: first in healthy specimens, second with a full thickness circular defect applied, and third time with the specimens repaired. Finally push out tests were performed to check whether the plug would remain in.

*Results*: Tests showed that applying a defect to the AF increases the range of motion (ROM), neutral zone (NZ) and neutral zone stiffness (NZS). In flexion/extension it was found significant for ROM, NZ, and NZS. For lateral bending and rotation a significant increase in ROM occurred. After AF repair ROM, NZ and NZS were normalized. All plugs remained in the AF during push out test up until 4000 N, but NP was squeezed out through the pores of the scaffold.

*Discussion*: A defect in the AF changes the biomechanical properties in the motion segment, changes that point to instability. Repairing the defect with a PCL plug and soft anchors brought the biomechanical behavior back to native state. This concept is promising and might be a viable way to repair the IVD after surgery.

## Introduction

Disc herniation is one of the most common spine disorders and cause of 300 000 surgeries annually in the U.S. [1]. While partial discectomy has good effect on acute leg pain, long-term results reveal a reoperation rate of approximately 25% [2] with many patients suffering from low back pain years after surgery [3]. This is a clear incentive to improve the existing surgical treatment.

Low back pain is a multifactorial disorder with changing mechanical conditions in the motion segments as one of the suggested etiologies [4,5]. The intervertebral disc (IVD) is an important component of spinal stability, and in case of degeneration, the motion segment becomes unstable [6]. When the annulus tears and disc herniation occur, the integrity of the annulus fibrosus (AF) and the segmental stability are further affected. An important goal in the treatment of a herniated disc must therefore be to restore the IVD function and stability of the motion segment.

Partial discectomy is a common treatment for disc herniation. But as the defect in AF is left untreated, reherniation can occur with more nucleus pulposus (NP) tissue being pushed through the defect. To avoid this, surgeons often remove as much of the NP as possible. This prevents reherniation but an important functional part of the IVD is lost and the stability of the motion segment is prone to further deterioration. Consequently, a viable approach to NP replacementor regeneration and restoration of the mechanical properties is heavily researched [7–9]. There is a consensus that in order to repair the IVD, the AF should be intact [10,11] lest the NP is squeezed out through the old defect.

An ideal solution would be to close the defect and restore stability simultaneously with removal of the herniation. This would prevent reherniation and on longer-term allow restoration of native AF tissue to enable replacement or regeneration of the NP. This requires a method that provides mechanical stability and allows for biointegration with AF. There can be advantages from using tissue-engineered scaffolds for AF repair. Scaffolds are often porous and designed to support cell migration and new tissue generation. Tissue engineering scaffolds allow for cell seeding and have the ability to release drugs [12,13].

This study uses a scaffold made from the polymer *polycaprolactone* (PCL), which is a well-known biomaterial and is hydrolytically degraded over a period of one or two years. It is used in several commercially available medical products.

This study investigated the ability to close an ex vivo herniation made in a porcine IVD with a scaffold and soft anchors and the impact on the motion segment movements.

## Materials and methods

### Test specimens

Nine porcine lumbar spine motion segments were used for testing the repair method. These specimens were cut out of spines harvested from three 40 kg pigs obtained from an abattoir. L1–L6 was cut into motion segments and soft tissue and muscles were removed leaving the posterior elements, pedicles, lamina, and facets intact. Motion segments' ends were embedded with low melting point bismuth alloy to mount in the testing machine. The annulus defect was made with a 3 mm biopsy punch (Miltex, Japan), excising a full thickness annulus biopsy. This left a 3 mm hole with the NP visibly bulging out when manipulated. NP was left in the disc.

### Closure

**Figure 1:**
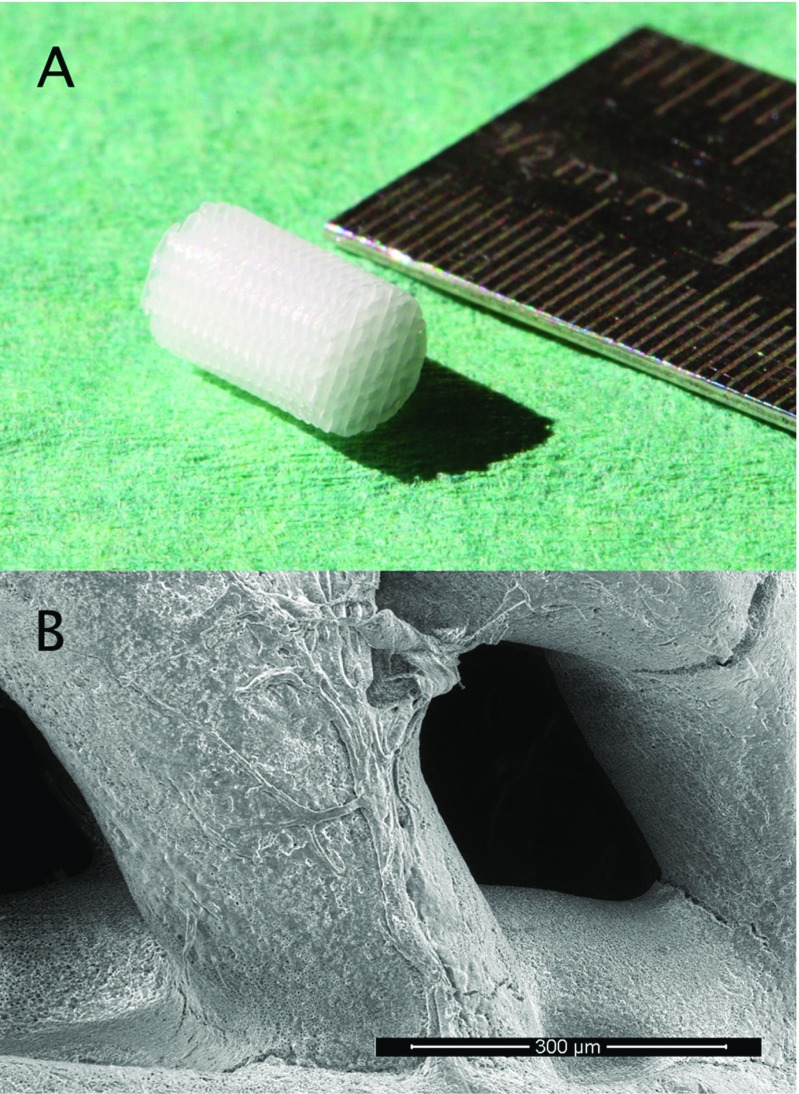
The scaffold, A: close up of the scaffold used for annulus fibrosus closure. B: SEM image of scaffold showing fiber surface and the porous design. Cell migration and survival and thus new tissue formation, is dependent on the scaffolds design features. These scaffolds were made on a 3D printer that allows control of fiber thickness, fiber pattern and pore size.

**Figure 2:**
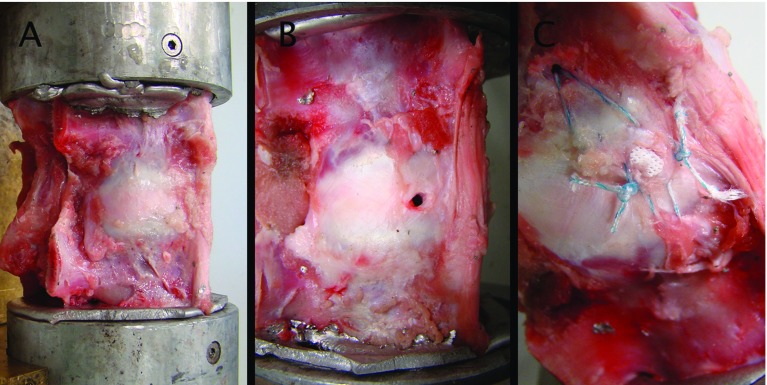
Each motion segment had the same test three times. A: in healthy native condition, B: with a 3 mm defect in the annulus fibrosus, C: after closing the defect with scaffold and soft anchors. Both ends are embedded in bismuth to insure rigid fixation.

A porous PCL scaffold was used as a plug for the circular defect (Figure 1). To close the defect, scaffolds were press fitted all the way into the defect, hence reaching 8 mm into AF and disc space. Next they were secured with soft anchors (Juggerknot 1.4 mm, Biomet) inserted in the vertebral body and sutured through the AF and scaffold (Figure 2). NP was left untouched in the disc space. The scaffold was made by 3D printing mats 200 µm fibers in layers consecutively angled 60 degrees on top of each to emulate the fiber direction of the AF. From these mats, individual scaffolds were cut out into cylinders 4 mm in diameter and 8 mm long using a biopsy punch.

To increase surface area, scaffolds were modified by partially dissolving the surfaces of the scaffold through immersion in 100% dioxane for 10 s followed by immersion in 80% ethanol/water to resolidify the polymer in a microporous arrangement. Finally, the scaffolds were treated with 5 M NaOH for 1 h to increase surface hydrophilicity by basic hydrolysis of the superficial polymer chains.

### Biomechanical testing

**Figure 3:**
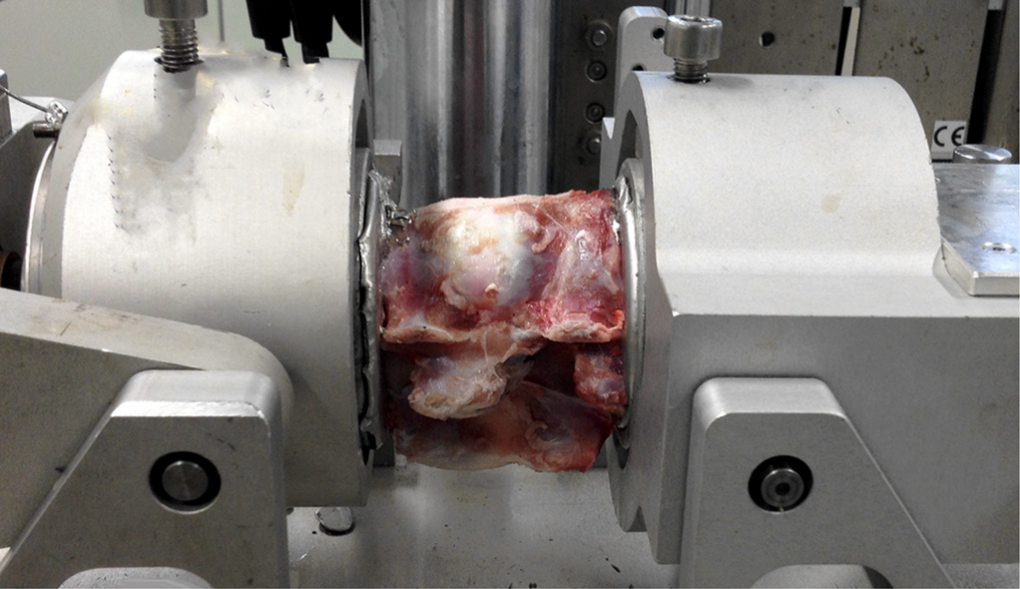
Test specimens were placed in a jig, seen on picture, that moves in 3 ways, flexion–extension, lateral bending and rotation. Load was applied by a servo hydraulic machine, which recorded load and displacement. By plotting load-displacement data, a sigmoid curve is drawn from where range of motion, neutral zone and neutral zone stiffness can be calculated.

**Figure 4:**
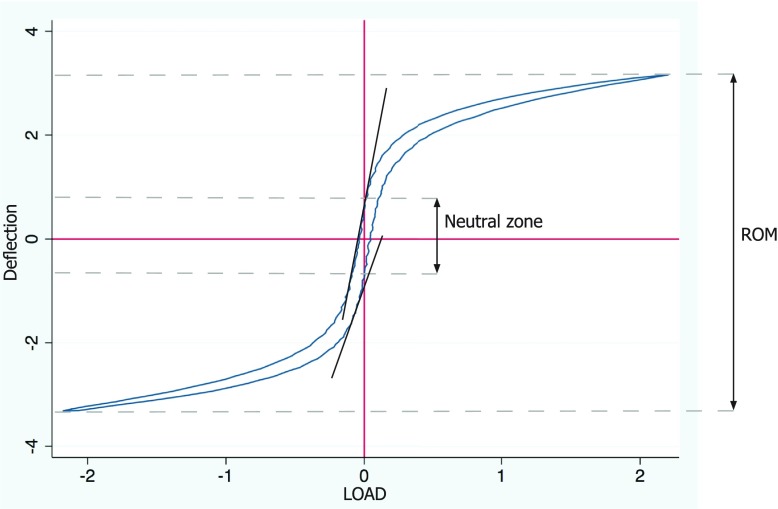
When load deflection data was plotted, two sigmoid curves were obtained, representing flextion and extension movement. Range of movement was measured as the maximum loading in each direction. Difference in intersection with the zero bar of load indicates the neutral zone. Neutral zone stiffness is the slope at zero load.

Each motion segment was tested three times − first time in native condition, second time with the defect simulating a herniation made in the antero-lateral part of the AF, and the third time with the defect repaired as described above. Biomechanical testing was done by placing the specimens in a custom made jig which allowed flexion/extension, bi-lateral bending and rotation (Figure 3). A maximum moment of 2 Nm was applied with a rate of 1 degree/s. To minimize the effect of hysteresis the motion segments went through ten loading cycles before data were obtained. Specimens were kept moist by covering with a saline soaked cloth. Finally, a push out test was performed applying axial compression until plug failure or 4000 N. For applying load and measuring displacement a servo hydraulic testing machine (Instron, Norwood, MA, USA) was used, acquiring data for all three directions and push out. Range of motion (ROM), neutral zone (NZ) and neutral zone stiffness (NZS) were obtained from load deflection data (Figure 4). NZ defined as region with minimal stiffness was measured as the angulation deference between flexion and extension at zero load. NZS was the slope at zero load.

The assumption of normality was checked by Q-Q plots, (Stata 12, Statacorp, USA). Groups were compared by means and Students *t*-test as a paired design. All *p*-values less than 0.05 were considered significant.

## Results

All plugs remained in the AF during the final push out test. A total axial pressure of 4000 N was applied in the axial direction with no failure. But, in all specimens NP leaking through the pores of the scaffold was observed. This happened at pressures ranging from 300 to 3100 N.

**Figure 5:**
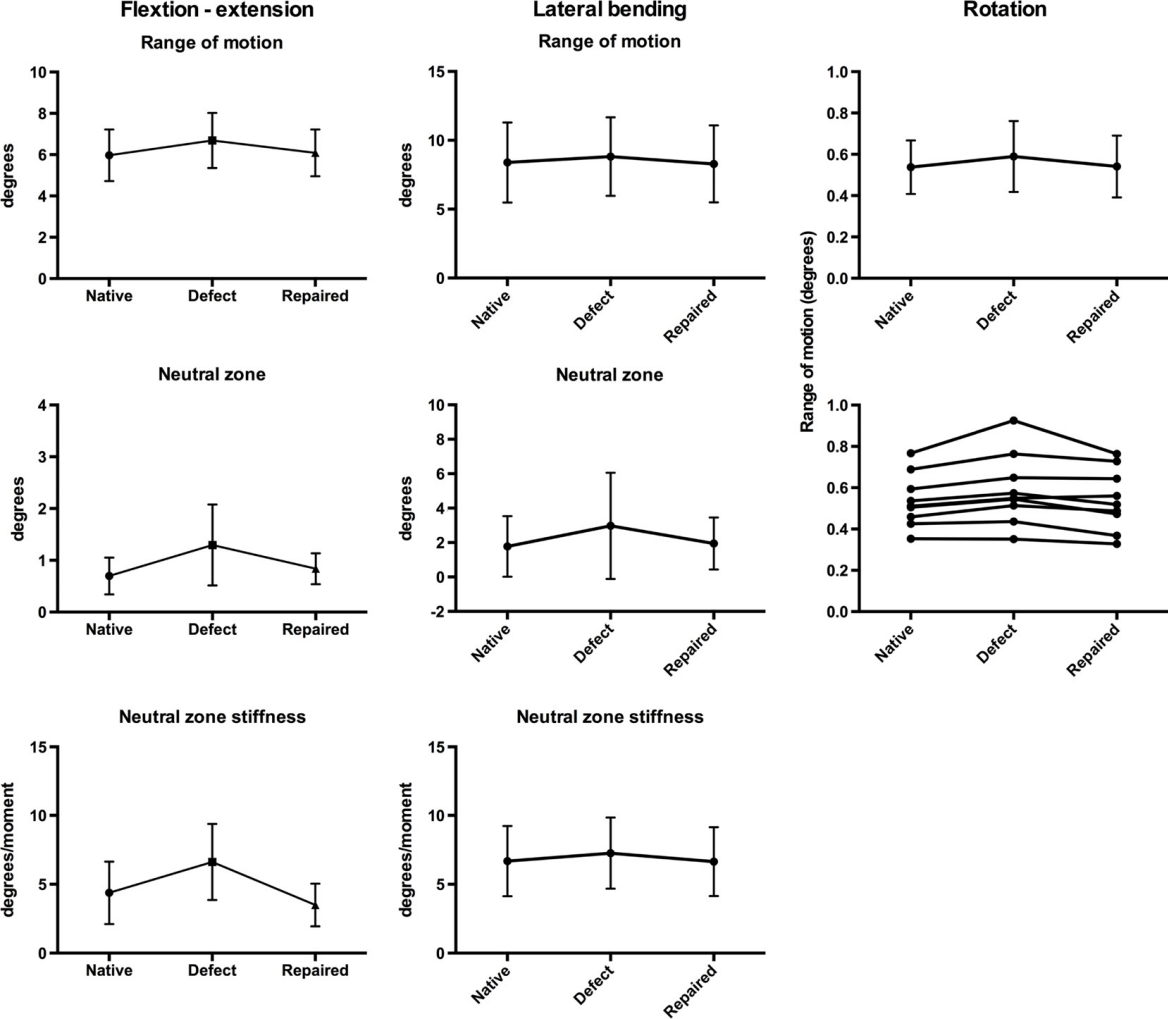
Graphs showing results of the 3 movements, flexion–extension, lateral bending and rotation. Means shown with standard deviations. Range of motion was increased when inducing a defect in AF and decreased when closing the defect, *p* < 0.0003. Neutral zone was also increased when applying the defect and decreased when closing it, but not significantly. Neutral zone stiffness also an indicator of stability in the motion segment showed deterioration when the AF was injured, but regained when the injury was closed.

Flexion/extension tests all showed increased ROM, NZ and NZS when applying the defect. Repairing the defect decreased the parameters again (Figure 5). This change was significant for ROM (*p* < 0.0003) and NZS (*p* < 0.004). The change in NZ was not significant (*p* < 0.09), though following the same pattern (Table 1).

**Table 1:** Mean values with confidence intervals − Flexion–extension.

	Native	Defect	Repaired
Range of motion	5.97(5.01; 6.93)	6.69(5.66; 7.71)	6.09(5.21; 6.96)
Neutral zone	0.70(0.42; 0. 97)	1.30(0.70; 1.90)	0.84(0.61; 1.07)
Neutral zone stiffness	4.38(2.64; 6.12)	6.62(8.71; 28.24)	6.98(3.53; 10.43)

Lateral bending showed the same pattern as flexion–extension, but only changes in ROM were found to be significant (*p* < 0.005) (Table 2).

**Table 2:** Mean values with confidence intervals − Lateral bending.

	Native	Defect	Repaired
Range of motion	8.39(6.15; 10.63)	8.82(6.62; 11.01)	8.29(6.14; 10.43)
Neutral zone	1.77(0.42; 3.13)	2.97(0.60; 5.34)	1.95(0.79; 3.11)
Neutral zone stiffness	6.68(4.72; 8.64)	7.26(5.28; 9.24)	6.64(4.72; 8.56)

Rotation showed a significant increase in ROM from 1.65(1.35; 1.96) to 1.81(1.41; 2.22), (*p* < 0.01) and was reduced again to 1.67(1.31; 2.02) (*p* < 0.02) when repaired (Table 3).

**Table 3:** Mean values with confidence intervals − Rotation.

	Native	Defect	Repaired
Range of motion	1.65(1.09; 2.36)	1.81(1.08; 2.85)	1.67(1.01; 2.35)

## Discussion

This study shows that a defect in the AF change the biomechanical properties of the motion segment, by increased ROM, NZ and NZS. It seems logical that when an IVD whose function relies on it to maintain pressure gets punctured, it loses stability. The impaired function of AF is likely to affect stability as well [6,14]. Closing the defect with a material with higher stiffness than the surrounding tissue, could possibly add to the rigidity in the motion segment. But, in this study, stability is regained when the defect is closed, and there is no significant difference between the healthy and the repaired motion segment. AF tissue has an elastic modulus around 0.42–0.82 MPa [15], but this scaffold has an elastic modulus about 6.8 MPa, which indicates more rigidity than the native AF. Although it did not to affect the motion segment stability, it might affect the discs ability to resist leaking. The purpose of using a scaffold is to promote tissue generation that resembles the native tissue. Fibrotic tissue, which has lower strength and elasticity, might occur anyway in some amount. This and integration of the more rigid scaffold in the AF will conceivably impair the resistance to the intradiscal pressure and a new herniation.

With exception of compression, no leakage was observed during lateral bending, flexion–extension or rotational testing, presumably due to the scaffold restoring some of the integrity of AF and thus helping to keep the pressure in the NP.

This ex vivo AF defect does not resemble a clinical herniation. The experimental set-up was designed to investigate the mechanical impact made by a defect in AF and how the repair worked. AF defects in clinical herniations can be very heterogeneous and the biomechanical properties of the IVD very different from those seen in a lab. This is one limitation that needs to be considered in this study and needs to be remembered when evaluating the AF repair method. Despite of this, previous studies have shown that the porcine spine is a very good approximation of the human spine, when it comes to anatomy and biomechanical properties [15,16]. The axial load on the IVD is most likely comparable [15]. Experiments have shown that the intradiscal pressure in humans can reach 2.3 MPa by lifting a 20 kg object with bent back [17]. In an experiment conducted by Bron et al. [18], an axial pressure of 600 N gave an intradiscal pressure of 3 MPa, in a similar set-up. In this experiment, the scaffold remained in the AF defect at loads up to 4000 N, but NP began to leak out at much lower loads.

Over the past few years, various methods for repairing AF have been examined experimentally both ex vivo and in vivo [18–21]. This is to our knowledge the first study to combine a plug and soft anchors in repairing the AF. Suturing and glue have been tested, but both the processes do not seem to have enough strength to withstand the mechanical load [22,23]. When closing a defect with only suture, tension is built up in the defect area as the suture is tightened. If the pressure inside a ring increases, it will increase the tension in the wall and the defect. This could result in failure of the repair. By doing the opposite, expanding the defect with a plug, presumably less tension will build up. This might prevent failure in closing the defect and also relieve pressure from other places in the annulus with poor condition. A caveat is that the plug will be expelled by the internal pressure in the disc if not secured in some way. Bron et al. examined polyethylene plugs with barbs in an in vivo study with goats. These were able to resist a compression force of 1000 N, but when used in vivo did damage to the endplates [18].

Mechanical tests of this scaffold showed an elastic modulus about 6.8 MPa, compared to hyaline cartilage of 0.5–0.9 MPa [24]. These values indicate a slightly harder plug than the native AF, but not likely to damage the endplates.

Since 3D printed scaffolds can be made with nearly complete design freedom, our approach shows great optimization opportunities. In future investigations, we will study the use of a differentiated pore sizes to keep NP inside while maintaining a compliance that matches that of the AF. NP replacement together with AF repair has to be tested as well.

This project was undertaken to design a scaffold for AF repair and evaluate its biomechanical function. The effect on spinal motion segment was increased stability. It did not manage to contain the NP in compression test, but did stay in the defect. Further research should focus on optimizing the scaffold and in vivo testing for evaluating biomechanical function and investigating biointegration.

## Conflicts of interest

The authors declare that they have no conflicts of interest in relation to this article.
